# Quantified reproductive isolation in *Heliconius* butterflies: Implications for introgression and hybrid speciation

**DOI:** 10.1002/ece3.3729

**Published:** 2017-12-20

**Authors:** Ivonne J. Garzón‐Orduña, Andrew V. Z. Brower

**Affiliations:** ^1^ Evolution and Ecology Group, Department of Biology Middle Tennessee State University Murfreesboro TN USA

**Keywords:** hybridization, introgression, reproductive isolation, speciation

## Abstract

*Heliconius* butterflies have become a model for the study of speciation with gene flow. For adaptive introgression to take place, there must be incomplete barriers to gene exchange that allow interspecific hybridization and multiple generations of backcrossing. The recent publication of estimates of individual components of reproductive isolation between several species of butterflies in the *Heliconius melpomene*–*H. cydno* clade allowed us to calculate total reproductive isolation estimates for these species. According to these estimates, the butterflies are not as promiscuous as has been implied. Differences between species are maintained by intrinsic mechanisms, while reproductive isolation of geographical races within species is mainly due to allopatry. We discuss the implications of this strong isolation for basic aspects of the hybrid speciation with introgression hypothesis.

## 1 INTRODUCTION

Since the demise of Ernst Mayr (1904–2005), emphasis on allopatry in the study of speciation has been eclipsed by ideas about ecological factors that might drive divergence of taxa in the absence of extrinsic barriers to reproduction (Jiggins, 2008; Rundle & Nosil, 2005; Schluter, 2009). The geographical component of speciation, once viewed as a key factor permitting the initial stages of divergence, which would otherwise be swamped by gene flow, is now often viewed as unnecessary, or at least passé. Various animals, including sticklebacks, timemas, and Darwin's finches, have become textbook icons exemplifying this shift of theoretical and empirical focus (Nosil, 2012).

*Heliconius* butterflies have likewise become a model system for studying patterns of speciation in the putative presence of ongoing gene flow (Dasmahapatra et al., 2012; Kronforst et al., 2006, 2013; Nadeau et al., 2013). Most *Heliconius* species are Müllerian mimics of one another, as well as other unpalatable taxa, displaying shared, aposematic wing patterns that are maintained by positive numerically dependent selection. Although selection would seem to favor a single, widespread signal to potential predators, paradoxically, multiple mimetic patterns may exist among different species occurring at a given locale, and further, many of these species also exhibit dramatic geographical variation in these wing patterns, so that the particular pattern shared between butterflies that mimic one another varies from place to place across their Neotropical distributions (Turner, 1975). While positive numerically dependent selection maintains this variability, it cannot explain its origin.

**Figure 1:**
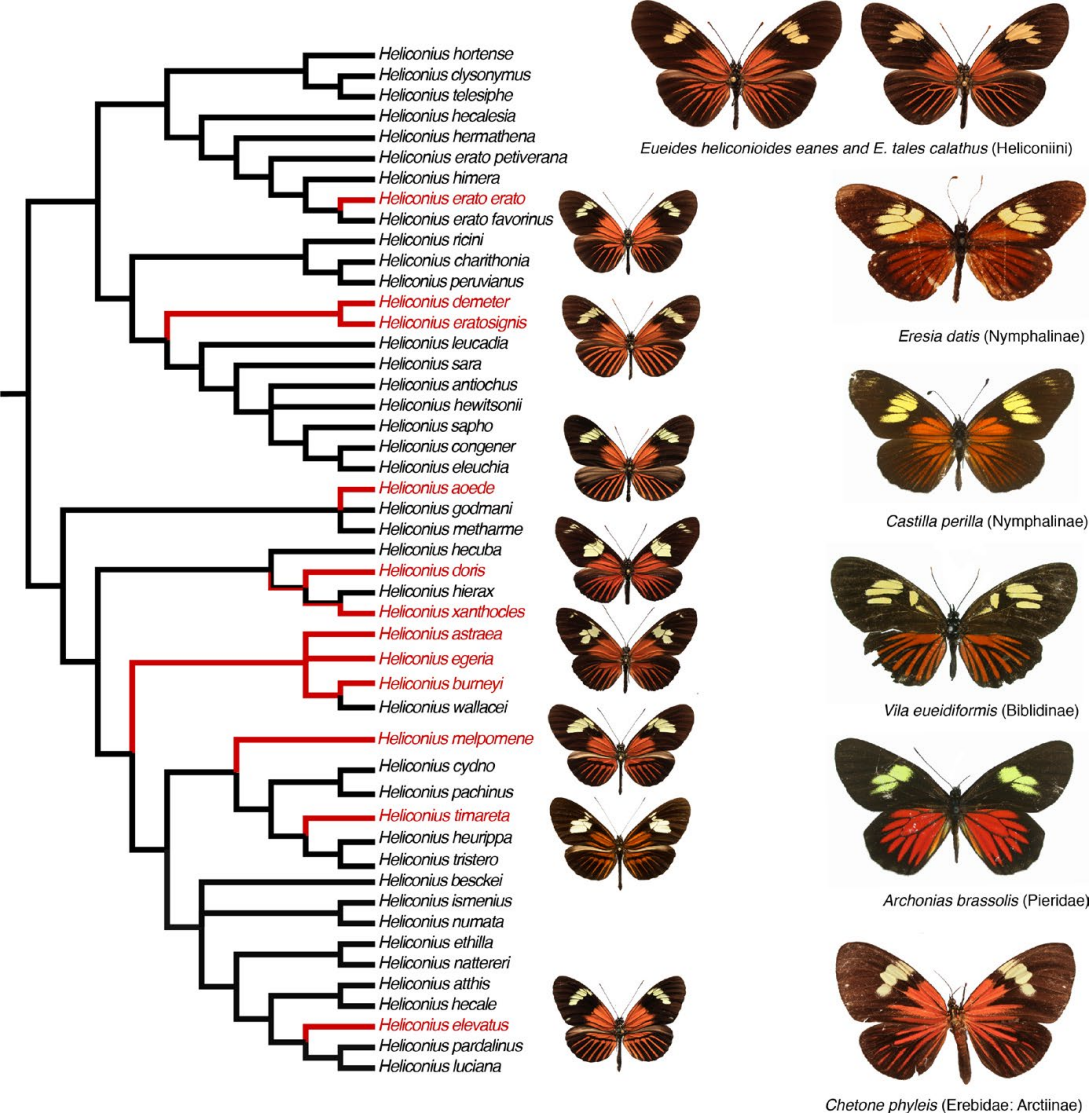
Multiple origins of the “dennis‐ray” mimetic pattern in *Heliconius* and other Lepidoptera. Cladogram of *Heliconius* species based on Brower and Garzón‐Orduña (2017). Exemplar *Heliconius* exhibiting the dennis‐ray pattern are illustrated (top to bottom, *H. erato*,* H. demeter*,* H. aoede*,* H. doris*,* H. burneyi*,* H. melpomene*,* H. timareta*,* H. elevatus*), and the origins of those features are parsimoniously optimized, indicating at least eight separate origins (red branches on tree and taxon labels). Note that *H. erato*,* H. melpomene,* and *H. timareta* also include geographical races that do not exhibit the dennis‐ray pattern. Exemplars of Müllerian or Batesian mimetic phenotypes of more distantly related butterflies and moths representing at least six further independent origins of the dennis and/or ray pattern are inset. Images are open access (Wallbank et al., 2016) or courtesy of Keith Willmott, Florida State Museum of Natural History

Traditionally, mimetic resemblance has been explained by convergent evolution, with similar wing patterns arising independently in separate lineages. It is clear, based on the diversity and phylogenetic distribution of particular aposematic patterns, that convergence remains the most plausible explanation for many instances of mimicry both within *Heliconius* and between the genus and its comimics (Figure 1). Thus, although convergence is a less parsimonious explanation for similarity than common ancestry, when mimetic wing patterns have evolved independently on multiple occasions within a taxon (not to mention in more remotely related groups—Figure 1), it is less onerous to explain shared patterns by that common mechanism than it is by invoking further, novel alternatives. One ad hoc hypothesis is more parsimonious than two ad hoc hypotheses.

Despite this, recent research has offered a new explanation for mimetic resemblance, suggesting that some *Heliconius* populations may have been able to shift from one mimicry ring to another by virtue of hybridization leading to adaptive introgression of mimetic wing pattern alleles and that one or more taxa may have arisen as a result of homoploid hybrid speciation (Dasmahapatra et al., 2012; Enciso‐Romero et al., 2017; Mávarez et al., 2006; Pardo‐Diaz et al., 2012). These hypotheses are counterintuitive, given the aforementioned selective regime that is thought to establish and maintain mimetic patterns in *Heliconius* communities. If positive numerically dependent selection favors abundant aposematic patterns, then if they could do so, why would not all potentially hybridizing species (not to mention actually hybridizing geographical races of the same species) converge upon the same mimetic pattern? One reason that wing pattern diversity is maintained could be that interspecific hybridization in *Heliconius* may not be as common and straightforward as has been advanced in the literature (Mallet, Beltrán, Neukirchen, & Linares, 2007).

According to the most recent checklist (Lamas & Jiggins, 2017), the melpomene‐cydno clade (Figure 1) comprises five species, three of which exhibit extensive diversification into phenotypically differentiated geographical races (Arias et al., 2014; Brower, 1996; Brown, 1979). These are *Heliconius melpomene*,* H. cydno*,* H. pachinus*,* H. timareta* (which now includes *H. tristero*, cf. Mérot et al., 2013), and *H. heurippa*. *Heliconius melpomene*, which generally exhibits red and yellow wing pattern elements, is widespread from Central America to southern Brazil, with numerous geographical races that are comimetic with sympatric forms of *H. erato*. *Heliconius melpomene* is the sister taxon to the other four species. *Heliconius cydno* occurs in Central America and west of the Andes as far south as Ecuador. Its color pattern elements are yellow and/or white on a black background. This species is also geographically differentiated into multiple races, comimetic with members of the *H. sapho* clade. *Heliconius pachinus* is a geographically restricted sister taxon of *H. cydno* that occurs in western Costa Rica, where it is a comimic of *H. hewitsoni*. *Heliconius timareta* replaces *H. cydno* east of the Andes, where it is now recognized to occur in at least seven geographically differentiated races that for the most part exhibit red and yellow pattern elements that mimic to a greater or lesser degree the sympatric races of *H. melpomene* and *H. erato* (Brower, 2011, 2013; Mallet, 2009). Nested allopatrically among these is *H. heurippa*, which occurs on the eastern slopes of the Andes in central Colombia, and although it has red and yellow forewing bands, is considered to be nonmimetic, as its wing pattern is not like that of any other *Heliconius* species. Although a consensus is beginning to develop that *H. heurippa* and *H. timareta* forms are conspecific (Arias et al., 2014; Brower, 2011; Jiggins, 2017; in which case all *H. timareta* forms should be considered subspecies of *H. heurippa* due to nomenclatural priority of publication), the traditional species names will be employed here.

Basic prerequisites for exchange of alleles between species are the success of interspecific mating events that produce F1 hybrids and the subsequent mating success of those hybrids with one or the other of the parental forms, resulting in introgression of alleles from one parental species into the other (Rieseberg & Wendel, 1993). It has long been known that mate discrimination in *Heliconius* is facilitated by visual recognition cues based on wing patterns (Crane, 1955; Jiggins, Naisbit, Coe, & Mallet, 2001; Merrill et al., 2011). This serves not only to promote conspecific, and to deter heterospecific mating in parental forms, but also to preclude mating opportunities for hybrid offspring (Naisbit, Jiggins, & Mallet, 2001). Further, interspecific mating experiments have shown that closely related species often produce sterile female offspring (Nijhout, Wray, & Gilbert, 1990; Salazar et al., 2004) following Haldane's Rule. Beyond these reproductive constraints, hybrid offspring with novel combinations of wing pattern elements that disrupt their participation in established mimicry rings also suffer strong selection due to predation (Mallet & Barton, 1989). All of these phenomena would seem to strengthen selective barriers to interspecific gene exchange (Brower, 2011).

Recently, Mérot, Salazar, Merrill, Jiggins, and Joron (2017) compiled published experimental data on the different components of reproductive isolation in the *Heliconius melpomene*–*H. cydno* clade*,* one of the groups in which introgression of wing pattern alleles between species has been hypothesized. They quantified various components of reproductive isolation due to pre‐ and postzygotic factors among many of the taxa in the clade, and provided some new evidence documenting the strength of reproductive isolation among members of this group. Surprisingly, however, Mérot et al. (2017) neglected to calculate total reproductive isolation, nor did they discuss their results in relation to the iconoclastic hypotheses described above. Given the clear implications of these data for the feasibility of speciation mechanisms that rely on adaptive introgression (cf. Jiggins, 2017), integrating these issues is desirable. Therefore, we take Mérot et al.'s results a step further, by calculating estimates of total reproductive isolation, and provide a quantitative summary that shows most of the species in the clade are completely or almost completely reproductively isolated from one another when the various components of isolation are combined. Finally, we discuss the plausibility of adaptive introgression in light of these reproductive constraints and other implications of the data that were not addressed in Mérot et al.'s publication.

## 2 METHODS

**Figure 2:**
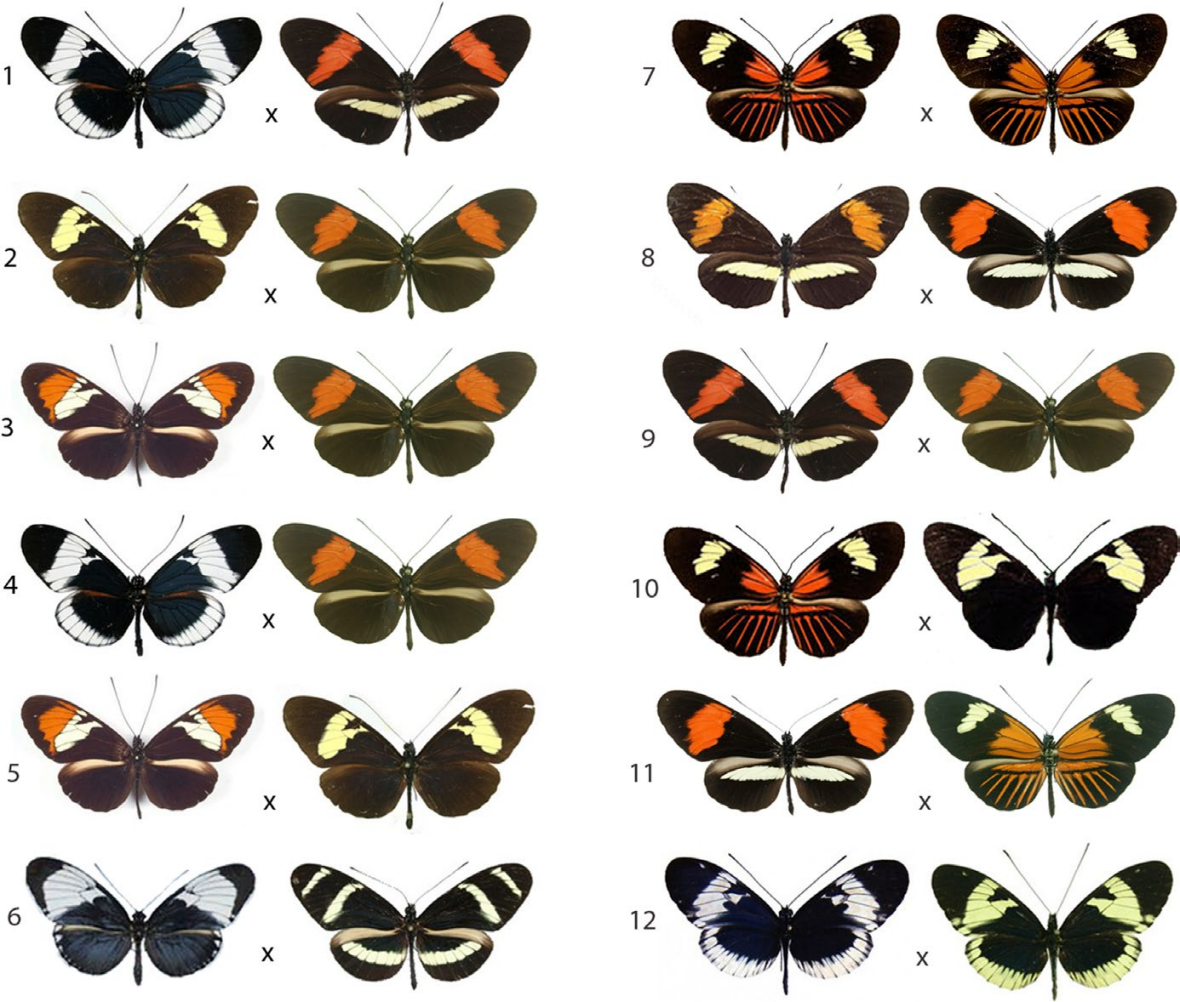
Wing patterns of crosses documented in Tables 1 and 2. Images from Brower (2013) or courtesy of Michel Cast (https://cliniquevetodax.com/Heliconius/index.html)

We used Mérot et al.'s (2017) estimates of individual isolation components (their Table 1) to calculate total reproductive isolation (TI) using the methods proposed by Sobel and Chen (2014). Briefly, Sobel and Chen suggested describing the relationship between the probability of gene flow and the probability of reproductive isolation as a linear equation that expresses the probability of reproductive isolation from 0 to 1, the former representing unrestricted gene flow (or disassortative mating) and the latter complete reproductive isolation (probability of gene flow under random mating is .5). Currently, data on the various components of reproductive isolation are available only for 12 matings involving various geographical races of the species described above; the comparisons examined here are based on these 12 mating pairs (Figure 2). The names and localities of these comparisons are given in Table 1. These comparisons range from interspecific (pairs 1–8), to between geographical races (pairs 9–11), and to sympatric polymorphic forms (pair 12).

**Table 1:** Locality data and references for the experimental crosses assessed

		Locality^a^	Coordinates, elevation	References
Pair	*H. cydno chioneus*	Pipeline Road, Panama	09°08′N, 79°42′W, 60 m	Naisbit, Jiggins, Linares, Salazar, Mallet (2002)
1	*H. melpomene rosina*	Pipeline Road, Panama	09°08′N, 79°42′W, 60 m	
Pair	*H. cydno cordula*	Barro Negro, Casanare, Colombia	06°01′06″N, 72°05′47″W, 1,050 m	Mérot et al. (2017)
2	*H. melpomene melpomene*	Río Charte, Casanare, Colombia	05°25′05″N, 72°31′20″W, 1,050 m	
Pair	*H. heurippa*	Villavicencio foothills, Colombia	04°07′N, 73°42′W, ~1,000 m	Mérot et al. (2017)
3	*H. melpomene melpomene*	Villavicencio foothills, Colombia	04°07′N, 73°42′W, ~1,000 m	
Pair	*H. cydno chioneus*	Pipeline Road, Panama	09°08′N, 79°42′W, 60 m	Naisbit et al. (2002)
4	*H. melpomene melpomene*	Pointe Macouria, French Guiana	04°58.4′N, 52°21.6′W, 0 m	
Pair	*H. heurippa*	Villavicencio foothills, Colombia	04°07′N, 73°42′W, ~1,000 m	Mávarez et al. (2006)
5	*H. cydno cordula*	Barro Negro, Casanare, Colombia	06°01′06″N, 72°05′47″W, 1,050 m	
Pair	*H. cydno galanthus*	La Selva, Costa Rica	10°03′N, 83°45′W, 2,000 m	Kronforst et al. (2006)
6	*H. pachinus*	Corcovado N.P., Costa Rica	08°27′N, 83°34′W, 22 m	
Pair	*H. timareta florencia*	Las Morres, Caquetá, Colombia	01°45′02″N, 75°37′55″W, 673–1,400 m	Mérot et al. (2017)
7	*H. melpomene malleti*	Las Morres, Caquetá, Colombia	01°45′02″N, 75°37′55″W, 673–1,400 m	
Pair	*H. timareta thelxinoe*	Alto Mayo, Tarapoto, Peru	05°39′58″S, 77°44′35″W 1,100–1,600 m	Mérot et al. (2015)
8	*H. melpomene amaryllis*	Alto Mayo, Tarapoto, Peru	05°39′58″S, 77°44′35″W 1,100–1,600 m	
Pair	*H. melpomene rosina*	Pipeline Road, Panama	09°08′N, 79°42′W, 60 m	Jiggins et al. (2001)
9	*H. melpomene melpomene*	Pointe Macouria, French Guiana	04°58.4′N, 52°21.6′W, 0 m	
Pair	*H. timareta florencia*	Las Morres, Caquetá, Colombia	01°45′02″N, 75°37′55″W, 673–1,400 m	Sanchez et al. (2015)
10	*H. timareta linaresi*	Guayabal, Caquetá, Colombia	02°41′04″N, 74°53′17″W, 1,350 m	
Pair	*H. melpomene amaryllis*	Tarapoto, Peru^b^	06°28′28″S, 76°20′35″W, 120 m	Merrill et al. (2011)
11	*H. melpomene aglaope*	Suniplaya, Peru^b^	05°57′28″S, 76°09′09″W, 138 m	
Pair	*H. cydno alithea white*	Mindo, Pichincha, Ecuador	02°42′S, 78°47′W, 1,375 m	Chamberlain, Hill, Kapan, Gilbert, Kronforst (2009)
12	*H. cydno alithea yellow*	Mindo, Pichincha, Ecuador	02°42′S, 78°47′W, 1,375 m	

Phenotypes are illustrated in Figure 2.

a In some instances, stocks for a given comparison were founded from specimens collected at more than one site. See the cited references for details.

b Data reported incorrectly by Mérot et al. (2017).

Sobel & Chen's calculation categorizes barriers to gene flow into three types: (extrinsic) prezygotic barriers that affect co‐occurrence, (intrinsic) prezygotic barriers not related to co‐occurrence, and postzygotic barriers (Sobel & Chen, 2014). As applied to the *Heliconius* species tested here, these variables reflect the degree of spatial overlap of the parental species, habitat preference, interspecific mating success, and the viability, mating success, and fertility of hybrid offspring. The values for the spatial component and mating were assigned to Sobel & Chen's first and second categories, respectively, while all the other variables were considered postzygotic barriers (F1 adult, F1 fertility, F1 egg, F1 mating with parent # 1, F1 mating with parent #2). We note, however, that the spatial co‐occurrence values of Mérot et al. (2017) reflected a conflation of allopatry in the traditional (geographical) sense and ecological habitat preference, such that species that appear to be sympatric according to range maps might have a deficit of encounters with each other due to relatively small altitudinal or ecological differences, such as larval host plant preference. For example, they reported a spatial isolation value of 0.74 between *H. melpomene rosina* and *H. cydno chioneus,* even though samples of both were collected along a few kilometers of Pipeline Road in Panama. As *Heliconius* butterflies are vagile animals that may move several kilometers over the course of their lives (Mallet et al., 1990), we view habitat preference within a geographical area as an intrinsic rather than an extrinsic barrier, and have separated these components accordingly in Table 2. Mérot et al. (2017) provided values for the strength of spatial isolation for only four of 12 interspecific mating comparisons, and we complemented these for the other eight species pairs based on distributional data regarding sympatry, parapatry, or allopatry (Brown, 1979).

**Table 2:** Components of reproductive isolation and total isolation as calculated by the formula of Sobel and Chen (2014)

	sp. 1—Female	sp. 2—Male	Spatial (allopatry)	Habitat preference	Mating	F1 eggs	F1 larvae	F1 adult	F1 fertility	F1 mating with sp. 1	F1 mating with sp. 2	Total rep. isolation reported by Mérot et al. (2017)^b^	Total isolation (TI) based on Sobel and Chen (2014)	TI excluding spatial component
Pair	*H. cydno chioneus*	*H. m. rosina*	0	0.74	1	0		0.35	0.32	0.2	0.52	100%	1	1
1	*H. m. rosina*	*H. cydno chioneus*	0	0.74	1	0		0.35	0.15				1	1
Pair	*H. cydno cordula*	*H. m. melpomene* VC	1^a^	n/a	0.82	0	0		0.29				1	0.8968
2	*H. m. melpomene*	*H. cydno cordula*	1^a^	n/a	0.88	0	0		0.18				1	0.9151
Pair	*H. heurippa*	*H. m. melpomene* VC	0	0.91	0.93	0	0		0.27	0.44	0.29		0.9996	0.9996
3	*H. m. melpomene*	*H. heurippa*	0	0.91	0.9	0	0		0.05	0.75	0.2		0.9996	0.9996
Pair	*H. cydno chioneus*	*H. m. melpomene* FG	1	n/a	0.78	0			0.48				1	0.9168
4	*H. m. melpomene*	*H. cydno chioneus*	1	n/a	1	0			0.34				1	1
Pair	*H. cydno cordula*	*H. heurippa*	1	n/a	0.56	0	0		0				1	0.56
5	*H. heurippa*	*H. cydno cordula*	1	n/a	0.98	0	0		0.07				1	0.9826
Pair	*H. cydno galanthus*	*H. pachinus*	0.9^c^	n/a	0.83					0	0.94		0.9995	0.9943
6	*H. pachinus*	*H. cydno galanthus*	0.9^c^	n/a	1					0	0.94		1	1
Pair	*H. t. florencia*	*H. m. malleti*	0	0.48	0.9	0			0.33			98%	0.9815	0.9815
7	*H. m. malleti*	*H. t. florencia*	0	0.48	0.96	0			0.19	0.52	1		1	1
Pair	*H. t. thelxinoe*	*H. m. amaryllis*	0	0.63	0.86	0	0		0.33	0.48	0	97%	0.994	0.994
8	*H. m. amaryllis*	*H. t. thelxinoe*	0	0.63	0.85	0	0		0.16	0.87	0		0.9981	0.9981
Pair	*H. m. melpomene* FG	*H. m. rosina*	1	n/a	1	0			0.07				1	1
9	*H. m. rosina*	*H. m. melpomene* FG	1	n/a	0.48	0			0.32				1	0.6935
Pair	*H. t. florencia*	*H. t. linaresi*	1	n/a	0.02	0			0.09				1	0.1
10	*H. t. linaresi*	*H. t. florencia*	1	n/a	0.48	0			0.09				1	0.5464
Pair	*H. m. aglaope*	*H. m. amaryllis*	0.9^c^	n/a	0.4								0.94	0.4
11	*H. m. amaryllis*	*H. m. aglaope*	0.9^c^	n/a	0								0.9	0
Pair	*H. c. alithea yellow*	*H. c. alithea white*	0	n/a	0.26			0.18			0.26		0.6133	0.6133
12	*H. c. alithea white*	*H. c. alithea yellow*	0	n/a	0.07			0.18			0.26		0.4763	0.4763

Component values are identical to those of Mérot et al. (2017), except for the spatial component (see Section 2).

a Although *H. melpomene melpomene* and *H. cydno cordula* are sympatric in parts of their ranges, these two samples are from allopatric populations.

b Reported by Mérot et al. (2017) in one direction only.

c Parapatric with hybrid zone.

All the calculations were performed using the supplementary Excel spreadsheet of Sobel & Chen (evo12362‐sup‐0003‐SuppMat.xls, equation RI_4E_). We reproduce the results obtained by Mérot et al. with the aforementioned modifications in Table 1, and present total reproductive isolation with and without consideration of the spatial component, which reflects the “actual” and “potential” aspects of reproductive isolation of the Biological Species Concept (Mayr, 1942). We emphasize that these values should be viewed as probabilities of reproductive isolation, not the inverse frequency or rate of interbreeding.

## 3 RESULTS AND DISCUSSION

Estimates of total reproductive isolation in Table 2 show that *Heliconius melpomene* and *H. cydno* are completely isolated in all comparisons when biogeography is taken into account (actual reproductive isolation), as are *H. cydno* and *H. pachinus*, and *H. cydno* and *H. heurippa*, and *H. melpomene* and *H. heurippa*. One cross, between *H. timareta florencia* females and *H. melpomene malleti* males, has a total isolation value of 0.9815. However, data for the backcross mating coefficient are lacking for this comparison. Backcross mating coefficients for the reciprocal cross of 0.52 and 1 were reported in the original study, and in that case, the pair is completely isolated. It is therefore likely that if comparable data had been included, *H. timareta florencia* females and *H. melpomene malleti* males would be completely isolated, as well. Also notably missing for ten of the twelve crosses is data for the survivorship of adult hybrids, which would also likely reduce the chances for backcrosses. If the experimental data reflect natural interactions between these species, available evidence indicates that the probability of interclade gene flow between *H. melpomene* and members of the cydno–heurippa–timareta species group is, from an absolute perspective, very small. This is corroborated by the extreme rarity, relative to intraspecific hybrids (hybrids between geographical races), of wild‐caught putative interspecific hybrid specimens in museum collections (Mallet et al., 2007).

When the biogeographical component is ignored in the calculations, reproductive isolation among allopatric conspecific forms decreases by an average of 27.5%, while it decreases by an average of only 0.7% for allopatric or parapatric heterospecific crosses. This suggests, in keeping with the “potential interbreeding” aspect of the Biological Species Concept, that allopatry appears to be a much more important component of the reproductive isolation for intraspecific crosses than it is for interspecific crosses (Coyne & Orr, 1989). That is, geographical races of *H. melpomene* behave as a single biological species, as do geographical races of *H. cydno* and *H. timareta*, respectively. *Heliconius pachinus* appears to be intrinsically isolated from *H. cydno,* as implied previously (Kronforst, Young, & Gilbert, 2007; Kronforst et al., 2006). In contrast, *H. heurippa* is only isolated from *H. cydno* by allopatry, suggesting that these two forms are parts of a single biological species (Brower, 2011). Experimental crosses testing isolation between *H. cydno* and *H. timareta* have not yet been reported.

Sympatric *H. melpomene* and *H. cydno* (pair 1) are more isolated than are allopatric *H. melpomene* and *H. cydno* (pairs 2 and 4), providing further evidence of reinforcement for conspecific mating fidelity between sympatric species, as found for adjacent versus remote populations of *H. cydno* and *H. pachinus* (Kronforst et al., 2007).

Although much has been made of the importance of color pattern in *Heliconius* mate recognition (Jiggins et al., 2001; Mávarez et al., 2006), the values for total isolation in Table 2 show that the two comimetic and visually almost identical, sympatric pairs of *H. melpomene* and *H. timareta* (pairs 7 and 8) are among the most isolated taxa in the dataset, and that a major contributing factor to this isolation is mate choice. This indicates that there must be additional cues, such as behavioral or pheromonal differences that allow species recognition/discrimination even when the butterflies look virtually the same (Mérot, Frérot, Leppik, & Joron, 2015). Nonvisual stimuli could serve both to enhance probabilities of intraspecific mating and to deter interspecific mating (Darragh et al., 2017; Friberg et al., 2008). Experiments testing only the visual component of mate choice using paper wing models, or using freshly emerged virgin females that may not have a full behavioral repertoire, are therefore not only artificial, but do not present a complete assessment of potential components of mate choice and/or discrimination. Thus, the “total isolation” values shown in Table 2 are likely to be underestimates of the actual isolation, when differential chemistry and behavior contribute to mate choice in nature. A further observation supporting the role of nonvisual cues in mate recognition is the fact that numerous hybrid zones exist within both *H. melpomene* and *H. cydno*, in which phenotypically different geographical races of the same species freely interbreed despite the fact that they have different wing patterns.

Of course, experimental evidence of “complete” reproductive isolation does not preclude the possibility of rare interspecific mating events. Through evolutionary time, extremely unlikely events can play a significant role. However, even if hybridization occurred occasionally, putatively beneficial introgressed alleles would need to survive several additional rounds of backcrossing to become integrated into the opposite genome. F1 and subsequent backcross offspring could suffer comparable pre‐ and postzygotic losses of fitness due to mate discrimination, sterility, and predation as measured above. If that is true, the quantitative values in Table 2 thus represent an overestimate of the very small likelihood of introgression.

The hypothesis stated by Mérot et al. (2017), “reproductive isolation between pairs at a high level of divergence is strong enough to allow the secondary loss of certain barriers to gene flow, in this case via the introgression of wing pattern alleles, without compromising genome‐wide differentiation,” may provide a plausible argument for the maintenance of mimicry, but not for its origin. By definition, if a species gains a new wing pattern via introgression, then it must have started with a different wing pattern than the one it displays today. The loss of the visual component of preexisting barriers to gene flow is a consequence of mimetic convergence, but necessitates that other barriers are strong enough to maintain species integrity, lest the two taxa undergo reverse speciation (Lackey & Boughman, 2016), and further implies that the species were even more strongly isolated from one another prior to the gene flow than they are now.

It has been suggested that *H. heurippa* and *H. timareta* forms have obtained their red pattern elements by introgression from *H. melpomene* (Giraldo, Salazar, Jiggins, Bermingham, & Linares, 2008; Mávarez et al., 2006; Pardo‐Diaz et al., 2012). The unparsimonious nature of the hybrid origin scenario for *H. heurippa* demonstrated by the reproductive isolation values reported here is compounded by the fact that now there are seven different geographical races of *H. timareta*, in which, given their phenotypic diversity and geographical distributions, the supposed introgression of different *H. melpomene* wing pattern alleles must have taken place independently at least five times. (Note that the underlying genetic capacity to produce red wing pattern elements in general is a symplesiomorphy for the entire melpomene–cydno clade—Figure 1.) Further, if introgression from *H. melpomene* is the means by which *H. timareta* has acquired its red wing pattern elements, then that mechanism fails to explain why *H. timareta timareta* is polymorphic with nonmimetic forms, and for that matter, why *H. heurippa* is not a comimic of its sympatric *H. melpomene* race (Brower, 2011).

**Figure 3:**
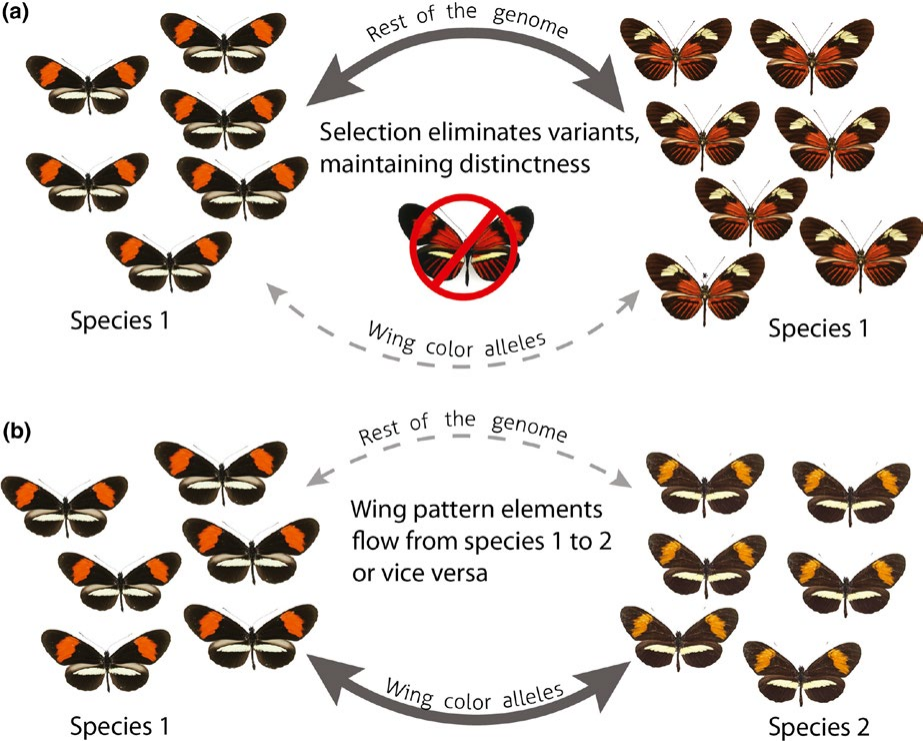
Contrasting patterns of gene flow and selection in (a) intraspecific hybrid zones within *Heliconius* species; and (b) interspecific hybridization between *Heliconius* species. Thickness of arrows indicates amount of gene flow of wing patterns and other alleles. In (a), introgression of wing pattern alleles is prevented by selection against novel hybrid phenotypes. In (b), according to the wing pattern introgression hypothesis, a different ancestral wing pattern has been replaced by introgressed, selectively advantageous wing pattern alleles without introgression of other loci. See text for details. Images courtesy of Michel Cast (https://cliniquevetodax.com/Heliconius/index.html)

If interspecific introgression of wing pattern alleles is a real phenomenon in *Heliconius*, then the interplay of selection and gene flow must be fundamentally different in cases of intraspecific versus interspecific hybridization. In intraspecific hybrid zones (e.g., the *H. melpomene amaryllis*–*H. melpomene aglaope* zone studied by Mallet & Barton, 1989; in the Huallaga Valley of Peru, Pair 11 in Table 2), there is no apparent mate discrimination based on wing pattern, and interracial mating takes place quite freely (Figure 3a). The two geographical races are maintained as distinct by very strong positive numerically dependent selection acting on those alleles, while alleles not related to wing pattern apparently mix readily (Turner, Johnson, & Eanes, 1979), such that there are “islands of divergence” in a sea of genetic homogeneity (Nadeau et al., 2012). In contrast, in interspecific crosses (Figure 3b), gene flow is minimal, due to the rarity of hybridization events, and wing pattern alleles become islands of similarity in a genomic sea that reflects underlying phylogenetic relationships (Enciso‐Romero et al., 2017). The apparently contradictory nature of selection in these cases remains a basic conundrum for the introgression hypothesis: If selection for Müllerian mimicry is strong enough to allow novel wing pattern alleles to flow across species boundaries that are virtually impenetrable (as the data here suggest), then why does wing pattern diversity persist among intraspecific forms of *H. melpomene*,* H. cydno,* and *H. timareta* that can freely interbreed among themselves?

Traditionally, the origin of *Heliconius* intraspecific wing pattern diversity has been explained by vicariance, perhaps due to the fragmentation of the rainforest during cool, dry Pleistocene climate cycles (Brown, 1979; Brown, Sheppard, & Turner, 1974). Molecular clock estimates for diversification of geographical races are consistent with this time frame (Brower, 1994; Garzón‐Orduña, Benetti‐Longhini, & Brower, 2014). Under this scenario, phenotypes of smaller, isolated populations could evolve novel wing patterns due to genetic drift (as in Phase 1 of the shifting balance; Wright, 1977), or be selected to converge upon wing patterns of other locally abundant unpalatable butterflies, such as members of the genera *Altinote*,* Melinaea,* or *Elzunia* (Turner & Mallet, 1996). Given the fact that all the current mimicry rings appear to have arisen relatively recently, it is possible that many other mimetic patterns may have existed in the past and gone extinct (cf. Linares, 1997). The strength of reproductive isolation found between sympatric species versus that between races separated only by geography is consistent with the hypothesis that the origin of novel wing patterns now maintained by intrinsic barriers was facilitated by allopatry.

Finally, we note that although the data on reproductive isolation reported by Mérot et al. (2017) and discussed here hardly characterize current species boundaries in the melpomene–cydno clade as a “continuum” (cf. Mallet et al., 2007), several objections might be posed. First, the mating experiments might measure unnatural behavior, or be insensitive to rare events that could allow gene flow to take place in spite of empirical “total isolation.” Or maybe the Sobel–Chen equation overestimates isolation. Third, perhaps contemporary strong reproductive isolation does not reflect the degree of isolation that may have existed over the past few hundred thousand years when purported introgressive hybridization events would have taken place. Of course, the past might not resemble the present. In our view, these are all ad hoc hypotheses to rescue a cherished theory from a parsimonious interpretation of the evidence, which suggests that interspecific transmission of precisely (and solely) those characters under selection to maintain specific differences is, at best, unlikely.

## CONFLICT OF INTEREST

None declared.

## AUTHOR CONTRIBUTIONS

IJGO and AVZB conceived the study, wrote the manuscript, and drafted the figures. IJGO ran the analyses.

## References

[ece33729-bib-0001] Arias, C. F. , Salazar, C. , Rosales, C. , Kronforst, M. R. , Linares, M. , Bermingham, E. , & McMillan, W. O. (2014). Phylogeography of *Heliconius cydno* and its closest relatives: Disentangling their origin and diversification. Molecular Ecology, 23, 4137–4152. https://doi.org/10.1111/mec.12844 24962067 10.1111/mec.12844

[ece33729-bib-0002] Brower, A. V. Z. (1994). Rapid morphological radiation and convergence among races of the butterfly *Heliconius erato* inferred from patterns of mitochondrial DNA evolution. Proceedings of the National Academy of Sciences of the United States of America, 91, 6491–6495. https://doi.org/10.1073/pnas.91.14.6491 8022810 10.1073/pnas.91.14.6491 PMC44228

[ece33729-bib-0003] Brower, A. V. Z. (1996). Parallel race formation and the evolution of mimicry in *Heliconius* butterflies: A phylogenetic hypothesis from mitochondrial DNA sequences. Evolution, 50, 195–221. https://doi.org/10.1111/j.1558-5646.1996.tb04486.x 28568874 10.1111/j.1558-5646.1996.tb04486.x

[ece33729-bib-0004] Brower, A. V. Z. (2011). Hybrid speciation in *Heliconius* butterflies? A review and critique of the evidence. Genetica, 139, 589–609. https://doi.org/10.1007/s10709-010-9530-4 21113790 10.1007/s10709-010-9530-4 PMC3089819

[ece33729-bib-0005] Brower, A. V. Z. (2013). Introgression of wing pattern alleles and speciation via homoploid hybridization in *Heliconius* butterflies: A review of evidence from the genome. Proceedings of the Royal Society of London B: Biological Sciences, 280, 20122302. 10.1098/rspb.2012.2302 PMC3574301 23235702

[ece33729-bib-0006] Brower, A. V. Z. , & Garzón‐Orduña, I. J. (2017). Missing data, clade support and “reticulation”: The molecular systematics of *Heliconius* and related genera (Lepidoptera: Nymphalidae) re‐examined. Cladistics (early view), https://doi.org/10.1111/cla.12198 10.1111/cla.12198 34645081

[ece33729-bib-0007] Brown, K. S. Jr (1979). Ecologia geográfica e evolução nas florestas neotropicais. Campinas, São Paulo: Universidade Estadual de Campinas.

[ece33729-bib-0008] Brown, K. S. Jr , Sheppard, P. M. , & Turner, J. R. G. (1974). Quaternary refugia in tropical America: Evidence from race formation in *Heliconius* butterflies. Proceedings of the Royal Society of London B: Biological Sciences, 187, 369–378. https://doi.org/10.1098/rspb.1974.0082

[ece33729-bib-0009] Chamberlain, N. L. , Hill, R. I. , Kapan, D. D. , Gilbert, L. E. , & Kronforst, M. R. (2009). Polymorphic butterfly reveals the missing link in ecological speciation. Science, 326, 847–850. https://doi.org/10.1126/science.1179141 19892982 10.1126/science.1179141 PMC2875868

[ece33729-bib-0010] Coyne, J. A. , & Orr, H. A. (1989). Patterns of speciation in *Drosophila* . Evolution, 43, 362–381. https://doi.org/10.1111/j.1558-5646.1989.tb04233.x 28568554 10.1111/j.1558-5646.1989.tb04233.x

[ece33729-bib-0011] Crane, J. (1955). Imaginal behavior of a Trinidad butterfly, *Heliconius erato hydara* Hewitson, with special reference to the social use of color. Zoologica (NY), 40, 167–196.

[ece33729-bib-0012] Darragh, K. , Vanjari, S. , Mann, F. , Gonzalez‐Rojas, M. F. , Morrison, C. R. , Salazar, C. , … Jiggins, C. D. (2017). Male sex pheromone components in *Heliconius* butterflies released by the androconia affect female choice. PeerJ, 5, e3953 https://doi.org/10.7717/peerj.3953 29134139 10.7717/peerj.3953 PMC5680698

[ece33729-bib-0013] Dasmahapatra, K. K. , Walters, J. R. , Briscoe, A. D. , Davey, J. W. , Whibley, A. C. , Nadeau, N. J. , … Jiggins, C. D. (2012). Butterfly genome reveals promiscuous exchange of mimicry adaptations among species. Nature, 487, 94–98. 22722851 10.1038/nature11041 PMC3398145

[ece33729-bib-0014] Enciso‐Romero, J. , Pardo‐Díaz, C. , Martin, S. H. , Arias, C. F. , Linares, M. , McMillan, W. O. , … Salazar, C. A. (2017). Evolution of novel mimicry rings facilitated by adaptive introgression in tropical butterflies. Molecular Ecology, 26, 5160–5172. https://doi.org/10.1111/mec.14277 28777894 10.1111/mec.14277

[ece33729-bib-0015] Friberg, M. , Vongvanich, N. , Borg‐Karlson, A.‐K. , Kemp, D. J. , Merilaita, S. , & Wiklund, C. (2008). Female mate choice determines reproductive isolation between sympatric species. Behavioral Ecology and Sociobiology, 62, 873–886. https://doi.org/10.1007/s00265-007-0511-2

[ece33729-bib-0016] Garzón‐Orduña, I. J. , Benetti‐Longhini, J. E. , & Brower, A. V. Z. (2014). Timing the diversification of the Amazonian biota: Butterfly divergences are consistent with Pleistocene refugia. Journal of Biogeography, 41, 1631–1638. https://doi.org/10.1111/jbi.12330

[ece33729-bib-0017] Giraldo, N. , Salazar, C. A. , Jiggins, C. D. , Bermingham, E. , & Linares, M. (2008). Two sisters in the same dress: *Heliconius* cryptic species. BMC Evolutionary Biology, 8, 324 https://doi.org/(310.1186/1471-2148-1188-1324) 19040737 10.1186/1471-2148-8-324 PMC2632674

[ece33729-bib-0018] Jiggins, C. D. (2008). Ecological speciation in mimetic butterflies. BioScience, 58, 541–548. https://doi.org/10.1641/B580610

[ece33729-bib-0019] Jiggins, C. D. (2017). The ecology and evolution of Heliconius butterflies. Oxford, UK: Oxford University Press.

[ece33729-bib-0020] Jiggins, C. D. , Naisbit, R. E. , Coe, R. L. , & Mallet, J. (2001). Reproductive isolation caused by colour pattern mimicry. Nature, 411, 302–305. https://doi.org/10.1038/35077075 11357131 10.1038/35077075

[ece33729-bib-0021] Kronforst, M. R. , Hansen, M. E. B. , Crawford, N. G. , Gallant, J. R. , Zhang, W. , Kulathinal, R. J. , … Mullen, S. P. (2013). Hybridization reveals the evolving genomic architecture of speciation. Cell Reports, 5, 666–677. https://doi.org/10.1016/j.celrep.2013.09.042 24183670 10.1016/j.celrep.2013.09.042 PMC4388300

[ece33729-bib-0022] Kronforst, M. R. , Young, L. G. , & Gilbert, L. E. (2007). Reinforcement of mate preference among hybridizing *Heliconius* butterflies. Journal of Evolutionary Biology, 20, 278–285. https://doi.org/10.1111/j.1420-9101.2006.01198.x 17210020 10.1111/j.1420-9101.2006.01198.x

[ece33729-bib-0023] Kronforst, M. R. , Young, L. G. , Kapan, D. D. , McNeely, C. , ONeill, R. J. , & Gilbert, L. E. (2006). Linkage of butterfly mate preference and wing color preference cue at the genomic location of wingless. Proceedings of the National Academy of Sciences of the United States of America, 103, 6575–6580. https://doi.org/10.1073/pnas.0509685103 16611733 10.1073/pnas.0509685103 PMC1458925

[ece33729-bib-0024] Lackey, A. C. R. , & Boughman, J. W. (2016). Evolution of reproductive isolation in stickleback fish. Evolution, 71, 357–372. 27901265 10.1111/evo.13114

[ece33729-bib-0025] Lamas, G. , & Jiggins, C. D. (2017). Taxonomic list In Jiggins C. D. (Ed.), The ecology and evoution of Heliconius butterflies (pp. 214–244). Oxford, UK: Oxford University Press.

[ece33729-bib-0026] Linares, M. (1997). The ghost of mimicry past: Laboratory reconstitution of an extinct butterfly ‘race’. Heredity, 78, 628–635. https://doi.org/10.1038/hdy.1997.102

[ece33729-bib-0027] Mallet, J. (2009). Rapid speciation, hybridization and adaptive radiation in the *Heliconius melpomene* group In Butlin R. K., Bridle J. R., & Schluter D. (Eds.), Speciation and patterns of diversity (pp. 177–194). Cambridge, UK: Cambridge University Press https://doi.org/10.1017/CBO9780511815683

[ece33729-bib-0028] Mallet, J. , & Barton, N. H. (1989). Strong natural selection in a warning‐color hybrid zone. Evolution, 43, 421–431. https://doi.org/10.1111/j.1558-5646.1989.tb04237.x 28568556 10.1111/j.1558-5646.1989.tb04237.x

[ece33729-bib-0029] Mallet, J. , Barton, N. , Lamas, G. , Santisteban, J. , Muedas, M. , & Eeley, H. (1990). Estimates of selection and gene flow from measures of cline width and linkage disequilibrium in *Heliconius* hybrid zones. Genetics, 124, 921–936. 2323556 10.1093/genetics/124.4.921 PMC1203983

[ece33729-bib-0030] Mallet, J. , Beltrán, M. , Neukirchen, W. , & Linares, M. (2007). Natural hybridization in heliconiine butterflies: The species boundary as a continuum. BMC Evolutionary Biology, 7, 28 https://doi.org/10.1186/1471-2148-7-28 17319954 10.1186/1471-2148-7-28 PMC1821009

[ece33729-bib-0031] Mávarez, J. , Salazar, C. A. , Bermingham, E. , Salcedo, C. , Jiggins, C. D. , & Linares, M. (2006). Speciation by hybridization in *Heliconius* butterflies. Nature, 441, 868–871. https://doi.org/10.1038/nature04738 16778888 10.1038/nature04738

[ece33729-bib-0032] Mayr, E. (1942). Systematics and the origin of species. New York, NY: Columbia University Press.

[ece33729-bib-0033] Mérot, C. , Frérot, B. , Leppik, E. , & Joron, M. (2015). Beyond magic traits: Multimodal mating cues in *Heliconius* butterflies. Evolution, 69, 2891–2904. https://doi.org/10.1111/evo.12789 26513426 10.1111/evo.12789

[ece33729-bib-0034] Mérot, C. , Mavarez, J. , Evin, A. , Dasmahapatra, K. K. , Mallet, J. , Lamas, G. , & Joron, M. (2013). Genetic differentiation without mimicry shift in a pair of hybridizing *Heliconius* species (Lepidoptera: Nymphalidae). Biological Journal of the Linnean Society, 109, 830–847. https://doi.org/10.1111/bij.12091

[ece33729-bib-0035] Mérot, C. , Salazar, C. , Merrill, R. M. , Jiggins, C. D. , & Joron, M. (2017). What shapes the continuum of reproductive isolation? Lessons from *Heliconius* butterflies. Proceedings of the Royal Society of London B: Biological Sciences, 284, 20170335 https://doi.org/10.1098/rspb.2017.0335 10.1098/rspb.2017.0335 PMC5474069 28592669

[ece33729-bib-0036] Merrill, R. M. , Gompert, Z. , Dembeck, L. M. , Kronforst, M. R. , McMillan, W. O. , & Jiggins, C. D. (2011). Mate preference across the speciation continuum in a clade of mimetic butterflies. Evolution, 65, 1489–1500. https://doi.org/10.1111/j.1558-5646.2010.01216.x 21521198 10.1111/j.1558-5646.2010.01216.x

[ece33729-bib-0037] Nadeau, N. J. , Martin, S. H. , Kozak, K. M. , Salazar, C. , Dasmahapatra, K. K. , Davey, J. W. , … Jiggins, C. D. (2013). Genome‐wide patterns of divergence and gene flow across a butterfly radiation. Molecular Ecology, 22, 814–826. https://doi.org/10.1111/j.1365-294X.2012.05730.x 22924870 10.1111/j.1365-294X.2012.05730.x

[ece33729-bib-0038] Nadeau, N. J. , Whibley, A. C. , Jones, R. T. , Davey, J. W. , Dasmahapatra, K. K. , Baxter, S. W. , … Jiggins, C. D. (2012). Genomic islands of divergence in hybridizing butterflies identified by large‐scale targeted sequencing. Philosophical Transactions of the Royal Society B: Biological Sciences, 367, 343–353. https://doi.org/10.1098/rstb.2011.0198 10.1098/rstb.2011.0198 PMC3233711 22201164

[ece33729-bib-0039] Naisbit, R. , Jiggins, C. D. , Linares, M. , Salazar, C. A. , & Mallet, J. (2002). Hybrid sterility, Haldane's rule and speciation in *Heliconius cydno* and *H. melpomene* . Genetics, 161, 1517–1526. 12196397 10.1093/genetics/161.4.1517 PMC1462209

[ece33729-bib-0040] Naisbit, R. E. , Jiggins, C. D. , & Mallet, J. (2001). Disruptive sexual selection against hybrids contributes to speciation between *Heliconius cydno* and *H. melpomene* . Proceedings of the Royal Society of London B: Biological Sciences, 268, 1849–1854. https://doi.org/10.1098/rspb.2001.1753 10.1098/rspb.2001.1753 PMC1088818 11522205

[ece33729-bib-0041] Nijhout, H. F. , Wray, G. A. , & Gilbert, L. E. (1990). An analysis of the phenotypic effects of certain color pattern genes in *Heliconius* (Lepidoptera: Nymphalidae). Biological Journal of the Linnean Society, 40, 357–372. https://doi.org/10.1111/j.1095-8312.1990.tb00545.x

[ece33729-bib-0042] Nosil, P. (2012). Ecological speciation. Oxford, UK: Oxford University Press https://doi.org/10.1093/acprof:osobl/9780199587100.001.0001

[ece33729-bib-0043] Pardo‐Diaz, C. , Salazar, C. , Baxter, S. W. , Merot, C. , Figueiredo‐Ready, W. , Joron, M. , … Jiggins, C. D. (2012). Adaptive introgression across species boundaries in *Heliconius* butterflies. PLoS Genetics, 8, e1002752 https://doi.org/10.1371/journal.pgen.1002752 22737081 10.1371/journal.pgen.1002752 PMC3380824

[ece33729-bib-0044] Rieseberg, L. H. , & Wendel, J. F. (1993). Introgression and its consequences in plants In Harrison R. G. (Ed.), Hybrid zones and the evolutionary process (pp. 70–109). Oxford, UK: Oxford University Press.

[ece33729-bib-0045] Rundle, H. D. , & Nosil, P. (2005). Ecological speciation. Ecology Letters, 8, 336–352. https://doi.org/10.1111/j.1461-0248.2004.00715.x

[ece33729-bib-0046] Salazar, C. A. , Jiggins, C. D. , Arias, C. F. , Tobler, A. , Bermingham, E. , & Linares, M. (2004). Hybrid incompatibility is consistent with a hybrid origin of Heliconius heurippa Hewitson from its close relatives, *Heliconius cydno* Doubleday and *Heliconius melpomene* Linnaeus. Journal of Evolutionary Biology, 18, 247–256. https://doi.org/10.1111/(ISSN)1420-9101 10.1111/j.1420-9101.2004.00839.x 15715831

[ece33729-bib-0047] Sanchez, A. P. , Pardo‐Diaz, C. , Enciso‐Romero, J. , Muñoz, A. G. , Jiggins, C. D. , Salazar, C. , & Linares, M. (2015). An introgressed wing pattern acts as a mating cue. Evolution, 69, 1619–1629. https://doi.org/10.1111/evo.12679 25930106 10.1111/evo.12679

[ece33729-bib-0048] Schluter, D. (2009). Evidence for ecological speciation and its alternative. Science, 323, 737–741. https://doi.org/10.1126/science.1160006 19197053 10.1126/science.1160006

[ece33729-bib-0049] Sobel, J. M. , & Chen, G. F. (2014). Unification of methods for estimating the strength of reproductive isolation. Evolution, 68, 1511–1522. https://doi.org/10.1111/evo.12362 24450287 10.1111/evo.12362

[ece33729-bib-0050] Turner, J. R. G. (1975). A tale of two butterflies. Natural History, 84, 28–37.

[ece33729-bib-0051] Turner, J. R. G. , Johnson, M. S. , & Eanes, W. F. (1979). Contrasted modes of evolution in the same genome: Allozymes and adaptive change in *Heliconius* . Proceedings of the National Academy of Sciences of the United States of America, 76, 1924–1928. https://doi.org/10.1073/pnas.76.4.1924 287032 10.1073/pnas.76.4.1924 PMC383505

[ece33729-bib-0052] Turner, J. R. G. , & Mallet, J. L. B. (1996). Did forest islands drive the diversity of warningly coloured butterflies? Biotic drift and the shifting balance. Philosophical Transactions of the Royal Society B: Biological Sciences, 351, 835–845. https://doi.org/10.1098/rstb.1996.0078

[ece33729-bib-0053] Wallbank, R. W. R. , Baxter, S. W. , Pardo‐Diaz, C. , Hanly, J. J. , Martin, S. H. , Mallet, J. , … Rull, J. (2016). Evolutionary novelty in a butterfly wing pattern through enhancer shuffling. PLoS Biology, 14, e1002353 https://doi.org/10.1371/journal.pbio.1002353 26771987 10.1371/journal.pbio.1002353 PMC4714872

[ece33729-bib-0054] Wright, S. (1977). Evolution and the genetics of populations. 3. Experimental results and evolutionary deductions. Chicago, IL: University of Chicago Press.

